# Socioeconomic factors from midlife predict mobility limitation and depressed mood three decades later; Findings from the AGES-Reykjavik Study

**DOI:** 10.1186/1471-2458-13-101

**Published:** 2013-02-04

**Authors:** Daniëlle AI Groffen, Annemarie Koster, Hans Bosma, Marjan van den Akker, Thor Aspelund, Kristín Siggeirsdóttir, Gertrudis IJM Kempen, Jacques ThM van Eijk, Gudny Eiriksdottir, Pálmi V Jónsson, Lenore J Launer, Vilmundur Gudnason, Tamara B Harris

**Affiliations:** 1 Department of Social Medicine, CAPHRI School for Public Health and Primary Care, Maastricht University, P.O. Box 616, 6200 MD Maastricht, The Netherlands; 2 Department of General Practice, School for Public Health and Primary Care (CAPHRI), Maastricht University, Maastricht, The Netherlands; 3 Department of General Practice, Katholieke Universiteit Leuven, Leuven, Belgium; 4 Icelandic Heart Association, Kopavogur, Iceland; 5 Faculty of Medicine, University of Iceland, Reykjavik, Iceland; 6 CAPHRI School for Public Health and Primary Care, Department of Health Services Research, Maastricht University, Maastricht, The Netherlands; 7 Laboratory of Epidemiology, Demography and Biometry, National Institute on Aging, National Institutes of Health, Bethesda, MD, USA

**Keywords:** Socioeconomic status, Mobility limitation, Depressed mood, Midlife, Old age

## Abstract

**Background:**

Taking into account our rapidly ageing population, older people are of particular interest in studying health inequalities. Most studies of older persons only include measures of current socioeconomic status (SES) and do not take into account data from earlier stages of life. In addition, only classic SES measures are used, while alternative measures, such as car ownership and house ownership, might equally well predict health. The present study aims to examine the effect of midlife socioeconomic factors on mobility limitation and depressed mood three decades later.

**Methods:**

Data were from 4,809 men and women aged 33–65 years who participated in the Reykjavik Study (1967–1992) and who were re-examined in old age in the Age, Gene/Environment Susceptibility (AGES) -Reykjavik Study (2002–2006).

**Results:**

Education and occupation predicted mobility limitation and depressed mood. Independently, home and car ownership and the availability of housing features predicted mobility limitation. Shortages of food in childhood and lack of a car in midlife predicted depressed mood.

**Conclusion:**

Socioeconomic factors from midlife and from childhood affect mobility limitation and depressed mood in old age. Prevention of health problems in old age should begin as early as midlife.

## Background

Socioeconomic health differences are consistent across different physical and mental health outcomes, countries and settings [1]. Taking into account our rapidly ageing population, older people are of particular interest in studying health inequalities. The origins of health inequalities in old age may not only be related to circumstances in old age, but may also be traced back to early life circumstances and experiences [2,3]. For example, low socioeconomic status (SES) in childhood is associated with higher morbidity in adulthood, independent of SES in adulthood [4,5].

In addition, there is an emphasis on classic SES measures (i.e. education, occupation, and income), while evidence exists that alternative measures, such as car ownership and house ownership, equally well predict health [6-8]. These measures may affect health via several pathways [7,9,10], independent of the classic SES measures. For example, the lack of a car may affect access to adequate health care and makes shopping for healthy foods more difficult. House ownership and lack of housing features are related to housing problems, overcrowding, and exposure to damp or cold and might consequently affect health [11]. Because the ownership of a car or a house might also be status symbols, psychosocial pathways can also not be excluded [12,13].

From a life course perspective [14], it is of interest to study the independent effects of different socioeconomic factors from earlier stages of life on old age functioning, excluding the possibility of recall bias [15]. In the present study, we use almost three decades of follow-up data from the Age, Gene/Environment Susceptibility (AGES)-Reykjavik Study to examine the independent effects of different socioeconomic factors from childhood and midlife on mobility limitation and depressed mood in old age.

## Methods

### Design and study population

The AGES-Reykjavik Study is drawn from the Reykjavik Study, a population-based cohort established in 1967 by investigators at the Icelandic Heart Association (IHA) [16,17]. This cohort originally comprised a random sample of 30,795 men and women born between 1907 and 1935, residing in Reykjavik, the capital of Iceland. A random sample of 27,281 persons was invited to participate and 19,381 individuals entered the study. As part of the Reykjavik study, participants completed questionnaires and participated in a standardized medical examination. Baseline measurements took place over a period of more than 20 years, from 1967 until 1992 [18].

The follow-up examination for AGES-Reykjavik started in 2002 by randomly selecting 8,030 individuals from the people who were still alive from the original Reykjavik-cohort (n = 11,459). A total of 5,764 individuals (58% women) entered the AGES-Reykjavik Study as participants. [18] The interval between the Reykjavik Study and the AGES-Reykjavik Study ranged from 13 to 38 years (mean = 28.8, SD = 5.6).

For the present study, persons older than 65 years at baseline (n = 109) or who had missing values on occupation (n = 107) or on one or more of the measures of health-related function (n = 431 for mobility limitation and n = 377 for depressed mood – partly overlapping persons) were excluded, leaving 4,809 participants for the current analyses. Forty-three percent of this population was men. At baseline, participants were aged between 33 and 65 years (mean age = 47.5, SD = 7.1). At follow-up, participants were aged between 66 and 93 years (mean age 76.3, SD = 5.32).

AGES-Reykjavik was approved by the National Bioethics Committee in Iceland that acts as the institutional review board for the IHA (approval number VSN-00-063) and the National Institute on Aging Intramural Institutional Review Board. A multistage consent is obtained for AGES–Reykjavik to cover participation and access to administrative records. Release of data for analysis is governed by rules created by these bodies to protect the privacy of Icelandic participants [18].

### Measures

#### Mobility limitation and depressed mood

Mobility limitation and depressed mood, as measured between 2002 and 2006, were used as indicators of physical and mental function, respectively. Mobility limitation was defined as report of any difficulty walking 500 meters or climbing 10 steps [19]. Depressed mood was measured using the 15-item version of the Geriatric Depression Scale (GDS) translated into Icelandic [20,21]. A score of five or higher was used to indicate depressive symptoms [22].

#### Socioeconomic factors

Two classic measures of SES were extracted from the midlife questionnaire. Educational level was defined as the highest level of completed education among which primary school or less, secondary education, college education, and university education. Because of small numbers in the third and fourth categories, college and university levels were combined so that three categories remained for the analyses. Occupation was categorized into four (study-specific) groups. The first group was designated as ‘homemaker/miscellaneous’. For men, this group included persons identified as having jobs that did not fall into any other category. For women, this first group included homemakers only. The second group, ‘manual’, included participants engaged in heavy manual work, e.g. dockworkers, fishermen, cement workers, and farm workers. Group three, ‘service workers’, consisted of participants engaged in light skilled crafts, clerical work and services, e.g. salesmen, postal clerks, typists, and nurses. Group four, ‘professional’, were participants engaged in non-physical work, such as directors, managers, teachers, doctors, and priests.

Alternative socioeconomic factors included house ownership (tenant or owner) and car ownership (yes/no). Participants also were asked to indicate whether their home included: central heating, a water closet, a bathtub, a shower, piped cold water, and piped hot water. Three categories were then created: possession of all of these features, lack of one feature, or lack of two or more features. For the logistic regression analyses group two and three were combined due to small numbers in the last category. Finally, during the follow-up examinations, respondents were asked if they had enough to eat when growing up (‘sometimes or often not enough to eat’ versus ‘mostly to often enough to eat’).

#### Covariates

Covariates were derived from the midlife questionnaire and included sex, age (33–65 years), year of first examination (1967–1992), marital status (currently single/currently together with a partner), and the presence of one or more chronic diseases (currently under doctor’s care or diagnosed with one or more of the following diseases: diabetes, angina pectoris, disease of the heart valves, coronary thrombosis, another heart disease, disease of the lungs, disease of the kidneys, disease of the thyroid, disease of the stomach/intestines, osteoarthritis, glaucoma, cancer, asthma or COPD, and tuberculosis).

### Statistical analyses

All analyses were performed using PASW version 19.0. Differences in main characteristics between men and women and educational levels were determined using chi-square tests for categorical variables and *t*-test and ANOVA based F-test statistics for continuous variables. Logistic regression analyses were performed to study whether socioeconomic factors were independently related to mobility limitation and depressed mood. The first model was adjusted for all covariates. The second model was additionally adjusted for all other socioeconomic factors. For all analyses, the most favorable socioeconomic position was used as a reference category.

## Results

Most socioeconomic factors were distributed in the expected direction (Table 1). Men and women with lower educational levels were more likely to report performing manual jobs, lacking one or more housing features, not having a car, not having enough to eat when growing up, and to report mobility limitation and depressed mood, as compared to men and women with higher educational levels. With regard to sex differences, level of education was significantly higher in men compared with women. Seventy-four percent of women reported themselves as homemakers at the time of first examination and 38% of men were engaged in heavy manual jobs. Women were more likely than men to report not owning a car and to report mobility limitation.

**Table 1:** Characteristics of the analysis sample, Reykjavik Study (1967–1992) and Age, Gene/Environment Susceptibility Study (2002–2006)

	**Men (N = 2068)**					**Women (N = 2741)**					**P-value**^**a**^
	**Total**	**Primary education N = 456 22.1%**	**Secondary education N = 1055 51.0%**	**College/ University** **N = 557 26.9%**	**p-value**^**a **^**education**	**Total**	**Primary education N = 1145 41.8%**	**Secondary education N = 1277 46.6%**	**College/ University** **N = 319 11.6%**	**p-value**^**a **^**education**	**men vs women P < 0.001**
Midlife factors											
Age, Mean (SD)	47.5(7.1)	47.1	46.6	46.6	0.367	48.1 (7.2)	48.8	47.4	48.8	0.567	<0.001 ^b^
≥1 chronic diseases ^c^ (%)	29.6	29.8	30.4	27.8	0.550	24.8	27.5	22.9	22.9	0.024	<0.001
Married/living together (%)	89.9	87.2	91.0	90.4	0.126	83.8	83.4	84.7	81.6	0.524	<0.001
Occupation (%)											
Homemaker/miscellaneous	4.2	8.1	3.9	1.6	<0.001	74.1	80.4	72.7	57.1	<0.001	<0.001
Manual	38.2	58.6	46.6	5.4		3.7	6.6	2.0	0.3		
Services	30.6	22.1	36.2	26.8		17.3	12.2	21.1	20.1		
Professional	27.1	11.2	13.3	66.2		4.9	0.8	4.2	22.6		
Did not own house (%)	10.8	13.8	10.2	9.5	0.061	11.1	14.2	8.8	8.5	<0.001	0.807
Did not personally own car (%)	13.1	21.3	11.2	10.1	<0.001	30.2	37.1	26.2	21.3	<0.001	<0.001
Housing features ^d^											
Lack of 2–6 features	3.7	8.1	3.1	1.1	<0.001	2.9	5.6	1.1	0.6	<0.001	0.265
Lack of 1 feature	44.3	53.1	45.0	35.9		45.7	53.3	42.2	32.6		
Possesses all features	52.0	38.8	51.8	63.0		51.4	41.1	56.7	66.8		
Early life circumstances (from AGES-Reykjavik)											
Not enough to eat (%)	5.2	8.8	4.7	3.2	0.001	2.8	4.3	2.0	0.9	0.001	<0.001
Old age outcome measures											
Mobility limitations ^e^ (%)	26.8	31.1	28.2	20.6	<0.001	39.5	43.8	37.7	32.0	<0.001	<0.001
Indication of depressed mood (%)	11.0	14.7	11.4	7.2	<0.001	12.8	14.8	12.3	7.2	0.001	0.058

^a^ Based on Chi-square tests for categorical variables and t-tests or ANOVA based F-tests for continuous variables; ^b^ This difference reflects the fact that women were recruited later; ^c^ Number of the following self-reported diseases at baseline: Diabetes, angina pectoris, disease of the heart valves, coronary thrombosis, another heart disease, disease of the lungs, disease of the kidneys, disease of the thyroid, disease of the stomach/intestines, arthritis, glaucoma, cancer, asthma or COPD, and tuberculosis; ^d^ Housing features include: central heating, water closet, bathtub, shower, piped cold water, and piped hot water; ^e^ Mobility limitation was considered to be present when a person reported any difficulty walking 500 meters or climbing 10 steps.

Excluded persons with missing values on physical dysfunction or depressed mood at follow up were significantly more likely to be older (p < 0.001), lower educated and to do heavy manual jobs (p < 0.001) at baseline. Similarly, persons with missing values on SES at baseline were more likely to report mobility limitation at follow up (p < 0.001) (data not shown).

Interaction terms between sex and any of the socioeconomic factors on mobility limitation and depressed mood were not significant (p > 0.10). Consequently, overall (not sex-specific) results of the logistic regression analyses are presented in Table 2. Men and women with primary or secondary education or engaged in heavy manual work had the highest risk of reporting mobility limitation and depressed mood in old age.

**Table 2:** Adjusted Odds Ratios (OR) of mobility limitation and depressed mood by socioeconomic factors; Reykjavik study (1967–1992) and Age, Gene/Environment Susceptibility Study (2002–2006)

	**Mobility limitation**				**Depressed mood**			
	**Model 1**^**a**^		**Model 2**^**b**^		**Model 1**		**Model 2**	
	**OR**	**95% CI**	**OR**	**95% CI**	**OR**	**95% CI**	**OR**	**95% CI**
Education								
Primary	1.62	1.34-1.96	1.49	1.21-1.84	2.11	1.56-2.84	1.87	1.35-2.58
Secondary	1.45	1.21-1.74	1.40	1.16-1.70	1.76	1.32-2.35	1.65	1.22-2.23
College/University	Ref.		Ref		Ref		Ref.	
Occupation								
Homemaker/Misc.	1.25	0.98-1.58	1.10	0.85-1.42	1.42	1.00-2.03	1.20	0.83-1.75
Manual	1.61	1.28-2.02	1.31	1.02-1.68	1.93	1.39-2.69	1.49	1.04-2.13
Services	1.31	1.05-1.64	1.16	0.92-1.47	1.29	0.92-1.81	1.10	0.78-1.55
Professional	Ref.		Ref		Ref.		Ref.	
Did not own house	1.26	1.03-1.53	1.15	0.94-1.40	1.25	0.96-1.64	1.13	0.86-1.50
Lack of ≥ 1 features	1.26	1.11-1.42	1.16	1.02-1.32	1.17	0.98-1.40	1.04	0.87-1.25
Did not own car	1.29	1.11-1.49	1.20	1.03-1.40	1.44	1.18-1.76	1.34	1.09-1.65
Not enough to eat	1.43	1.05-1.95	1.34	0.98-1.83	1.78	1.22-2.60	1.63	1.11-2.39

^a^ Adjusted for sex, age at baseline, year of first examination, marital status, and number of chronic diseases; ^b^ Additionally adjusted for all other socioeconomic factors.

Most other socioeconomic factors were related to an increased risk of mobility limitations and depressed mood. Even when adjusted for each other, lacking one or more housing features and not owning a car were significant predictors of mobility limitation. Not owning a car and reporting not to have enough to eat as a child were independent predictors of depressed mood three decades later.

Additional adjustment for behavioral factors (i.e. BMI, physical activity and smoking status), did not significantly change the results (data not shown).

## Discussion

We were able to examine data from a cohort of 4,809 men and women with an average of 29 years of follow-up. Men and women from lower educational levels had the highest risk of mobility limitation and depressed mood almost three decades later. Independently, car ownership and the availability of housing features were predictors of mobility limitation. Recalled shortages of food in childhood and midlife car ownership were additional predictors of depressed mood.

Our findings support the hypothesis that various socioeconomic factors from childhood and midlife independently predict mobility limitation and depressed mood in old age. The independent effects of low educational and occupational level, not owning a car, lack of housing features, and shortages of food might imply different mechanistic pathways in which these factors affect health. While education relates to differences between people in terms of access to information and making (healthy) lifestyle choices, the possession of resources and the living environment may have direct effects on physical health. For example, through exposure to damp and cold, and access to fresh food [8,23]. In addition, psychological pathways cannot be excluded either. Thus, instead of considering material factors such as car and home ownership, housing features and lack of food as ‘alternative’ measures of SES, or as mediators in the relation between SES and health, these measures could be an important, maybe the most toxic, part of the concept ‘SES’ itself.

Our findings also support the hypothesis that not only exposures during adulthood, but in early life as well are important for health in old age [3,14]. In our study, recall of not having enough food in childhood emerged as an independent predictor of depressed mood in old age [3,14]. The prevention of disability in old age should, at least, begin already in midlife (and perhaps even earlier) [3]. From a public health perspective, more research on this issue is needed to help develop adequate (environmental) interventions.

### Strengths and limitations

The strengths of this study include a long follow-up period and the possibility to study the influence of various socioeconomic factors as measured in midlife. This study also has some limitations. First, no data on income was available which could have led to a potential for confounding by income in our analyses. For example, car and home ownership might be dependent upon income. Previous research by Macintyre and colleagues (1998), however, showed that housing tenure and car access were significant predictors of health, even after adjustment for income [7]. Also in our analyses, housing features and car ownership remained significant predictors of mobility limitation and depressed mood after adjustment for income-proxies, such as occupation and education.

Second, we were not able to exclude persons with prevalent physical dysfunction and depressed mood at baseline, however, all analyses were adjusted for the prevalence of chronic diseases at baseline. Third, socioeconomic factors and covariates were assessed only at baseline and these baseline data were collected over an unusually long period (20+ years). Socioeconomic factors could have easily changed during this time period. It is, however, unclear how these changes might have affected the results of our study. Finally, our research may be limited by potential selection biases. Excluded respondents (n = 955; described in the Methods section) were, for example, more likely to be older (p < 0.001), lower educated (p < 0.001), to report the lack of a car (p < 0.001), and not to own the house (p < 0.001), as compared to included respondents. Moreover, previous results from the Reykjavik Study [24] indicated that people from lower educational level were more likely to die prematurely. Consequently, the most disadvantaged persons may be underrepresented in the AGES-Reykjavik Study. This pattern of selective response and attrition may have led to an underestimation of the reported associations.

## Conclusion

Our findings support the hypothesis that socioeconomic factors from midlife and even childhood affect mobility limitation and depressed mood in old age. Prevention of health problems in old age should thus begin as early as midlife. The independent effects of socioeconomic factors imply multiple starting points for action. More research is needed to help develop adequate environmental interventions.

## Abbreviations

AGES: Age, Gene/Environment, Susceptibility Study; PASW: Predictive analytics software (formerly known as SPSS); ANOVA: Analysis of variance; 95% CI: 95% confidence interval; OR: Odds ratio; SES: Socioeconomic status; SD: Standard deviation; BMI: Body mass index; GDS: Geriatric depression scale.

## Competing interest

The authors declare that they have no competing interests.

## Authors’ contributions

DG performed the statistical analyses and drafted the manuscript. AK helped to interpret the data. DG and HB formulated the hypotheses. TBH, VG, PVJ and LJL were responsible for the design of the AGES-Reykjavik Study. GE and TA were responsible for data management of the AGES-Reykjavik study. KS, PVJ were responsible for data acquisition and data preparation. All authors (DG, AK, HB, MvdA, TA, KS, GIJMK, JThMvE, GE, PVJ, LJL, VG, TBH) edited and approved the final manuscript.

## Pre-publication history

The pre-publication history for this paper can be accessed here:

http://www.biomedcentral.com/1471-2458/13/101/prepub
